# Preoperative TIPS prevents the development of postoperative acute-on-chronic liver failure in patients with high CLIF-C AD score

**DOI:** 10.1016/j.jhepr.2022.100442

**Published:** 2022-01-21

**Authors:** Johannes Chang, Pauline Höfer, Nina Böhling, Philipp Lingohr, Steffen Manekeller, Jörg C. Kalff, Jonas Dohmen, Dominik J. Kaczmarek, Christian Jansen, Carsten Meyer, Christian P. Strassburg, Jonel Trebicka, Michael Praktiknjo

**Affiliations:** 1 Department of Internal Medicine I, University Hospital Bonn, Bonn, Germany; 2 Center for Cirrhosis and Portal Hypertension Bonn (CCB), University Hospital Bonn, Bonn, Germany; 3 Department of Visceral Surgery, University Hospital Bonn, Bonn, Germany; 4 Department of Radiology, University Hospital Bonn, Bonn, Germany; 5 Translational Hepatology, Department of Internal Medicine 1, University Hospital Frankfurt, Frankfurt, Germany; 6 European Foundation for the Study of Chronic Liver Failure, Barcelona, Spain

**Keywords:** Surgery, Acute decompensation, TIPS, Cirrhosis, ACLF, Acute-on-chronic liver failure, Transjugular intrahepatic portosystemic shunt, ACLF, acute-on-chronic liver failure, AD, acute decompensation, ASA, American Society of Anesthesiologists, CLIF-C AD, Chronic Liver Failure Consortium–Acute Decompensation, HE, hepatic encephalopathy, HR, hazard ratio, HVPG, hepatic venous pressure gradient, MELD, model for end-stage liver disease, ROC, receiver-operation characteristics, TIPS, transjugular intrahepatic portosystemic shunt, VOCAL, Veterans Outcomes and Costs Associated With Liver Disease

## Abstract

**Background & Aims:**

Acute-on-chronic liver failure (ACLF) is a syndrome associated with organ failure and high short-term mortality. Recently, the role of surgery as a precipitating event for ACLF has been characterised. However, the impact of preoperative transjugular intrahepatic portosystemic shunt (TIPS) placement on ACLF development in patients with cirrhosis undergoing surgery has not been investigated yet.

**Methods:**

A total of 926 patients (363 with cirrhosis undergoing surgery and 563 patients with TIPS) were screened. Forty-five patients with preoperative TIPS (TIPS group) were 1:1 propensity matched to patients without preoperative TIPS (no-TIPS group). The primary endpoint was the development of ACLF within 28 and 90 days after surgery. The secondary endpoint was 1-year mortality. Results were confirmed by a differently 1:2 matched cohort (n = 176).

**Results:**

Patients in the no-TIPS group had significantly higher rates of ACLF within 28 days (29 *vs*. 9%; *p* = 0.016) and 90 days (33 *vs.* 13%; *p* = 0.020) after surgery as well as significantly higher 1-year mortality (38 *vs*. 18%; *p* = 0.023) compared with those in the TIPS group. Surgery without preoperative TIPS and Chronic Liver Failure Consortium–Acute Decompensation (CLIF-C AD) score were independent predictors for 28- and 90-day ACLF development and 1-year mortality after surgery, especially in patients undergoing visceral surgery. In the no-TIPS group, a CLIF-C AD score of >45 could be identified as cut-off for patients at risk for postoperative ACLF development benefiting from TIPS.

**Conclusions:**

This study suggests that preoperative TIPS may result in lower rates of postoperative ACLF development especially in patients undergoing visceral surgery and with a CLIF-C AD score above 45.

**Lay summary:**

Acute-on-chronic liver failure (ACLF) is a syndrome that is associated with high short-term mortality. Surgical procedures are a known precipitating event for ACLF. This study investigates the role of preoperative insertion of a transjugular intrahepatic portosystemic shunt (TIPS) on postoperative mortality and ACLF development. Patients with TIPS insertion before a surgical procedure exhibit improved postoperative survival and lower rates of postoperative ACLF, especially in patients undergoing visceral surgery and with a high CLIF-C AD prognostic score. Thus, this study suggests preoperative TIPS insertion in those high-risk patients.

## Highlights

• This study investigates the impact of preoperative TIPS on postsurgical ACLF.
• Patients with preoperative TIPS, especially before visceral surgery, develop significantly lower rates of ACLF.
• Preoperative TIPS is associated with improved postsurgical survival.
• CLIF-C AD score >45 can be used as cut-off for patients at risk for postsurgical ACLF.
• Selected patients might benefit from preoperative TIPS insertion.

## Graphical abstract

**Figure:**
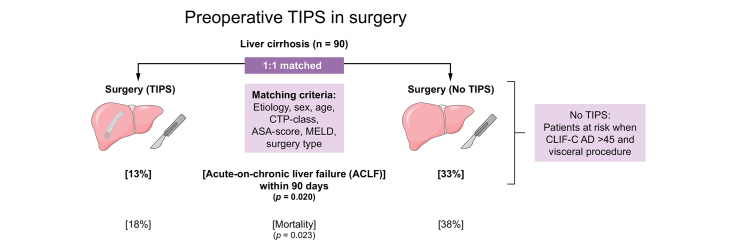

## Introduction

Cirrhosis is the common end stage of chronic liver diseases and is characterised by fibrosis of liver tissue, decrease in liver function, and the development of portal hypertension.^1^^,^^2^ Acute decompensation (AD) such as refractory ascites and acute variceal bleeding can be treated with placement of transjugular intrahepatic portosystemic shunt (TIPS) in selected patients.[3], [4], [5], [6] However, AD can progress to acute-on-chronic liver failure (ACLF), a syndrome defined by the development of multi-organ failure resulting in high short-term mortality.[7], [8], [9]

Severe alcoholic hepatitis, proven bacterial infections, and variceal bleeding are the most common precipitating events for ACLF,^1^^,^^10^ but many suggested precipitants have not been sufficiently studied yet. Recently, the role of surgery as a precipitating event for ACLF development has been characterised, confirming high rates of ACLF development after surgical procedures.^11^

Until recently, TIPS placement itself has been discussed as a precipitating event for the development of ACLF.^12^ However, current literature indicates that placement of TIPS in eligible patients has a rather beneficial effect concerning further development of ACLF episodes and other liver-related endpoints, probably attributed to reduction of clinically significant portal hypertension. An observational multicentre study suggested that placement of TIPS in patients with ACLF and acute variceal bleeding improves survival and rebleeding rates.^13^ In the context of surgery, our group could show a negligible precipitating effect of TIPS placement for the development of ACLF compared with surgical interventions.^14^

Moreover, recent data show that high hepatic venous pressure gradients (HVPGs) of >16 and ≥20 independently predicted 90-day and 1-year mortality in patients undergoing elective extrahepatic surgery.^15^ These data indicate a potential benefit of TIPS insertion before surgery by reduction of the portosystemic pressure gradient. Data concerning this hypothesis are scarce, and thus, the question of whether preoperative TIPS placement has an effect on the postoperative development of ACLF or mortality, remains unanswered. Therefore, this retrospective study aimed to investigate the impact of preoperative TIPS placement on ACLF development and mortality in patients with liver cirrhosis undergoing surgery.

## Patients and methods

### Patients and data collection

In this retrospective single-centre study, patients with cirrhosis undergoing surgery (no-TIPS group) were 1:1 propensity matched and compared with patients with cirrhosis undergoing surgery but with preoperative TIPS placement (TIPS group). The primary endpoint of this study was the development of ACLF within 28 and 90 days. The secondary endpoint was 1-year mortality.

For the identification of the study cohort, a total of 926 patients with liver cirrhosis between July 2006 and December 2019 of the Department of Internal Medicine I, University of Bonn, Germany, were screened. At the time of surgery, all screened patients were ≥18 years old and had clinical, radiological, or histological findings to confirm liver cirrhosis.

#### Identification of patients for the no-TIPS group

**Fig. 1:**
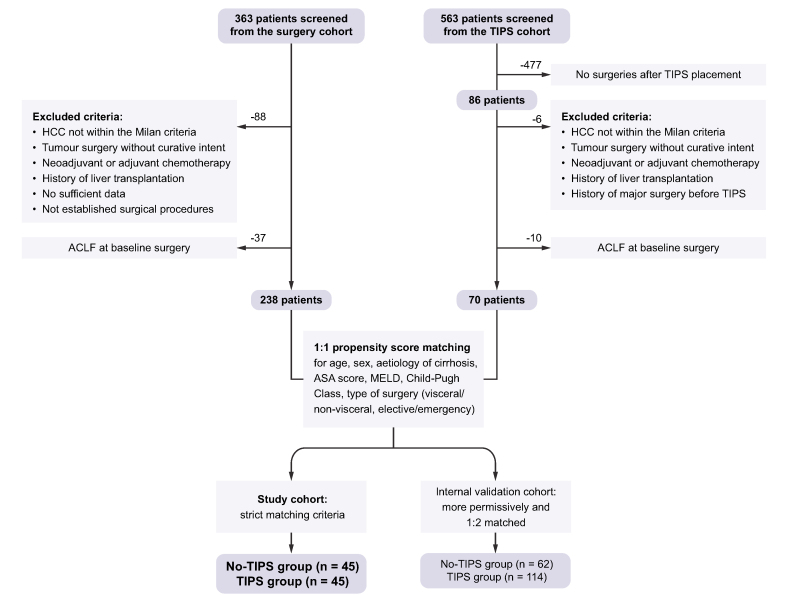
Diagram depicting the selection and matching processes to define the final study cohort of patients with cirrhosis and preoperative TIPS (TIPS cohort) *vs*. patients with cirrhosis undergoing surgery without preoperative TIPS (no-TIPS cohort). ACLF, acute-on-chronic liver failure; ASA, American Society of Anesthesiologists; HCC, hepatocellular carcinoma; MELD, model for end-stage liver disease; TIPS, transjugular intrahepatic portosystemic shunt.

A total of 363 consecutive patients with cirrhosis who had undergone a surgical procedure between July 2006 and December 2017 were screened. These patients were identified using hospital database search based on the International Statistical Classification of Diseases and Related Health Problems, 10th Revision (ICD-10), as previously described.^11^ Eighty-eight patients undergoing surgical procedures owing to hepatocellular carcinoma not within the Milan criteria, non-standardised surgical procedures, and surgery with palliative intent or requiring adjuvant or neoadjuvant chemotherapy as well as patients without sufficient data or with a history of liver transplantation at the time of surgery were excluded. Further, 37 patients who presented with ACLF at the time of surgery were excluded, resulting in 238 patients eligible for propensity matching (Fig. 1).

#### Identification of patients for the TIPS group

Out of 563 consecutive patients from the observational NEPTUN (Non-invasive Evaluation Program for TIPS and Follow Up Network; clinicaltrials.gov identifier: NCT03628807) cohort that had undergone TIPS placement between September 2008 and December 2019, 86 patients who had a relevant surgical procedure after TIPS placement were identified. Of those, 6 patients undergoing surgical procedures owing to hepatocellular carcinoma not within the Milan criteria or surgery with palliative intent or requiring adjuvant or neoadjuvant chemotherapy and patients with a history of liver transplantation at the time of surgery or a history of major surgery before TIPS placement were excluded. Ten further patients who presented with ACLF at baseline were excluded. Thus, 70 patients were eligible for propensity matching (Fig. 1). Indications for TIPS insertion were refractory ascites or variceal bleeding. None of the TIPS insertions were performed pre-emptively to surgery.

### Propensity score matching

Patients from the no-TIPS group were 1:1 propensity matched with patients from the TIPS group. Matching criteria were aetiology of cirrhosis, sex, Child-Pugh class, type of surgery (visceral/non-visceral and emergency/elective surgery), American Society of Anesthesiologists (ASA) score, model for end-stage liver disease (MELD) ± 3 points, and age ±3 years. Forty-five patients from the no-TIPS group were propensity score matched with 45 patients from the TIPS group. This final cohort of 90 patients was enrolled in the analyses (Fig. 1). Twenty-five patients of the TIPS cohort remained unmatched; the cohort characteristics of these patients are shown in Table S1. For internal validation with less excluded patients and increased statistical power, an additional 1:2 (TIPS *vs*. no-TIPS) propensity score matching with more permissive matching criteria was performed including 176 patients (n = 62 in the TIPS group and n = 114 in the no-TIPS group).

### Data collection

Patient medical records were used to obtain clinical and laboratory data before and after surgery. ACLF was diagnosed retrospectively according to the EASL-Chronic Liver Failure (CLIF) ACLF criteria.^7^ To define organ failures of ACLF within the 90-day follow-up period, the Chronic Liver Failure Consortium–Sequential Organ Failure Assessment (CLIF-C-SOFA) score was retrospectively applied as suggested in current EASL guidelines.^1^ Respiratory failure was diagnosed when mechanical ventilation was required for reintubation or reasons other than airway protection exceeding the standard postoperative care in the absence of hepatic encephalopathy (HE) grade III or IV. Arterial hypotension (mean arterial pressure below 70 mmHg) or the use of vasopressors with an indication other than hepatorenal syndrome therapy was classified as circulatory failure. Postoperative ascites development was defined as the need for paracentesis, as the observation of ascites in postoperative abdominal drainage, or based on imaging findings within 90 days after surgery. Overt HE was defined clinically using West Haven criteria within 90 days after the surgical procedure.^1^

### Statistical analysis

For all variables, descriptive statistics were performed. Non-parametric testing was used to compare the groups. Propensity score matching of the no-TIPS and TIPS groups was performed by using the MatchIt (version 3.0.2) package in R (R Foundation for Statistical Computing, Vienna, Austria). Survival rates were analysed using Kaplan–Meier curves with the log-rank test. Univariate and multivariate Cox regression analysis with stepwise forward selection was used to identify predictors of ACLF development within 28 and 90 days after surgery. Significant parameters in univariate regression analysis and known risk factors were entered in multivariate regression analyses. Scores (*e.g.* Chronic Liver Failure Consortium–Acute Decompensation [CLIF-C AD] score or MELD) were not simultaneously entered with their respective components to avoid collinearity. Sensitivity analysis as published by Ding and Van der Weele^16^^,^^17^ was performed for the multivariate models. The prognostic value and selection of optimal cut-off values according to the Youden index for CLIF-C AD and MELD were analysed using receiver operating characteristics (ROC) with 90-day ACLF development as the endpoint.

Values of *p* <0.05 were considered to be statistically significant. Continuous variables are presented as median and range. Categorical variables are presented as absolute cases or percentage. All data were analysed using SPSS (version 24, IBM, Armonk, NY, USA) and R (version 4.0.2), augmented by R Studio (version 1.3.1073, RStudio, Inc., Boston, MA, USA).

## Results

### General patient characteristics

Ninety patients (45 from the no-TIPS group 1:1 matched with 45 from the TIPS group) were included in the analyses. Because of strict matching for confounders, there were no differences in sex, aetiology, and Child-Pugh class between the groups. In both groups, patients were predominantly male (n = 32 [71%] in each group) and were mostly categorised into Child-Pugh class B (n = 35 [78%] in each group) at baseline. The most frequent cause of cirrhosis was alcohol-related liver disease (n = 32 [71%] in each group) (Table 1). More than half of the surgical procedures in both groups were visceral (n = 24 [53%] in each group) (Table 1 and Table S2A). Surgery types between the 2 groups did not differ significantly, especially the number of liver resections (5 [11%] in the TIPS group *vs*. 6 [13%] in the no-TIPS group, *p* = 0.749) (Table S2B). Patients were mostly classified with an ASA score of 3 (n = 35 [78%] in each group). The median age was similar between the TIPS and no-TIPS groups (63 [43–80] and 64 [40–77] years, respectively, *p* = 0.54). The median of CLIF-C AD score was not statistically different between the TIPS and no-TIPS groups at baseline (46 [29–64] *vs*. 49 [28–61], respectively, *p* = 0.74), and MELD and liver-related laboratory parameters did not show any significant differences (Table 1). The distribution of the presence of varices and grades of varices before surgery (for the TIPS group before TIPS placement) was not significantly different between the 2 groups. After TIPS placement, the presence of varices was lower in the TIPS group. Sodium, platelet count, and spleen size as surrogate parameters for portal hypertension were comparable between the 2 groups. None of the patients presented with overt HE at the time of surgery (Table 1). The indication for TIPS placement was refractory ascites in 30 (67%) patients and acute variceal bleeding in 15 (33%) patients. None of the patients showed signs of TIPS dysfunction at the time of surgery. Two patients presented with controlled ascites at surgery, both with TIPS placement only within 1 month before surgery. The median time between TIPS placement and surgery was 6 (0–101) months.

**Table 1:** **General characteristics of 1:1 matched patient cohort: 45 patients with TIPS *vs.* 45 patients without TIPS (n = 90)**.

Parameters at baseline	TIPS (n = 45)	No TIPS (n = 45)	*p* value
General conditions			
Age (years)	63 (43–80)	64 (40–77)	0.54
Sex (male/female)	32/13 (71/29%)	32/13 (71/29%)	1.00
Aetiology (alcohol/viral hepatitis/other)	32/4/9 (71/9/20%)	32/4/9 (71/9/20%)	1.00
BMI	25.8 (17.4–34)	25.5 (18–35)	0.93
Baseline scores			
MELD score	11 (6-17)	10 (6–18)	0.32
Child-Pugh class A/B	10/35 (22/78%)	10/35 (22/78%)	1.00
CLIF-C AD score	46 (29–64)	49 (28–61)	0.74
Baseline laboratory			
Sodium (mmol/L)	140 (130–145)	139 (130–145)	0.20
Potassium (mmol/L)	4.14 (2.93–5.16)	4.07 (3.24-5.3)	0.76
Creatinine (mg/dl)	1.07 (0.49-1.76)	0.85 (0.46–1.86)	0.07
Bilirubin (mg/dl)	1.28 (0.32-4.85)	1.09 (0.24-3.85)	0.41
ALT (U/L)	24 (9–80)	23 (7-83)	0.65
AST (U/L)	38 (18-278)	39 (11–155)	0.82
Albumin (g/dl)	32 (20.8–46.2)	30 (22.4-42)	0.21
GGT (U/L)	63 (50-79)	66 (15-82)	0.83
Alkaline phosphatase (U/L)	135 (69–349)	150 (13–523)	0.72
INR	1.2 (1-1.6)	1.2 (1–2.2)	0.93
CRP (mg/L)	9.2 (0.48–58.6)	12.6 (0.7–60.3)	0.27
Hb (g/dl)	10.5 (8–16)	10.9 (7.5–16.3)	0.19
WBC (10^3^/μl)	5.16 (2.25–11.77)	5.84 (1.23–11.64)	0.25
Platelets	122.5 (25–336)	137 (23–394)	0.27
Baseline clinical conditions			
Ascites	2(4%)	7(16%)	0.10
Varices before surgery (before TIPS) grade I/II/III	11/13/11 (24/29/24%)	14/13/5 (31/29/11%)	0.42
Varices before surgery (after TIPS) grade I/II/III	6/3/0 (13/7/0%)	14/13/5 (31/29/11%)	0.00
HE	0 (0%)	0 (0%)	1.00
Spleen diameter (cm)	14 (10–26)	14.5 (8.5–22.8)	0.54
Use of rifaximin	1 (2%)	2 (4%)	0.56
Surgery			
Non-visceral/visceral	21/24 (47/53%)	21/24 (47/53%)	1.00
Emergency/elective	7/38 (16/84%)	7/38 (16/84%)	1.00
ASA score (1/2/3/4)	1/4/35/5 (2/9/78/11%)	1/4/35/5 (2/9/78/11%)	1.00
Medical history			
History of ascites	35 (77.8%)	31 (68.9%)	0.34
History of GI bleeding	18 (40%)	13 (29%)	0.27
History of HE	8 (17.8%)	7 (15.6%)	0.78

Data are shown as median and ranges. Non-parametric testing was used to compare the groups, Mann-Whitney U test for comparison between continuous variables and Chi-squared test for comparison between categorical variables.

ALT, alanine transaminase; ASA, American Society of Anesthesiologists; AST, aspartate transaminase; CLIF-C AD, Chronic Liver Failure Consortium–Acute Decompensation; CRP, C-reactive protein; GGT, gamma glutamyl-transferase; GI, gastrointestinal; Hb, haemoglobin; HE, hepatic encephalopathy; INR, international normalised ratio; MELD, model for end-stage liver disease; TIPS, transjugular intrahepatic portosystemic shunt; WBC, white blood cells.

The more permissively 1:2 matched validation cohort included 176 patients (62 with TIPS and 114 without TIPS). The clinical characteristics of the validation cohort are shown in Table S3.

### Characteristics of postoperative ACLF

**Fig. 2:**
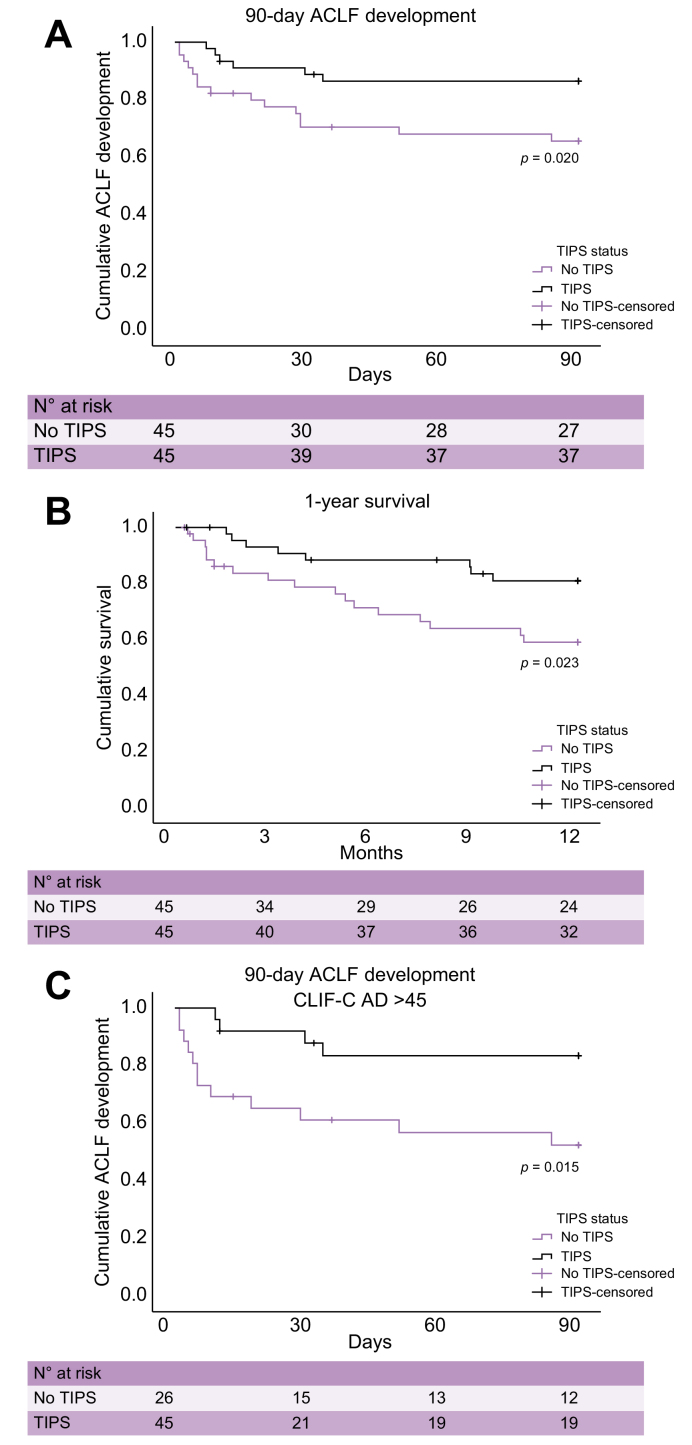
Kaplan–Meier plots showing mortality and probability of 90-day ACLF development for patients undergoing surgery in the TIPS and no-TIPS groups. (A) Probability of 90-day ACLF development calculated according to the log-rank test for patients in the TIPS and no-TIPS groups (n = 90). Level of significance *p* = 0.020. (B) One-year survival calculated according to the log-rank test for patients in the TIPS and no-TIPS groups (n = 90). Level of significance *p* = 0.023. (C) Probability of 90-day ACLF development calculated according to the log-rank test for patients with a CLIF-C-AD >45 stratified to the TIPS and no-TIPS groups (n = 51). Level of significance *p* = 0.015. ACLF, acute-on-chronic liver failure; CLIF-C AD, Chronic Liver Failure Consortium–Acute Decompensation; TIPS, transjugular intrahepatic portosystemic shunt.

Kaplan–Meier analysis shows significantly lower rates of postoperative ACLF for the TIPS group within 28 days after surgery than those for the no-TIPS group (n = 4 [8.9%] *vs*. n = 13 [28.9%], *p* = 0.013) (Fig. S1). Similarly, within 90 days after surgery, patients in the TIPS group developed significantly less ACLF than those in the no-TIPS group (n = 6 [13%] *vs*. n = 15 [33%], *p* = 0.020) (Fig. 2A). The same results could be shown in the more permissively matched validation cohort (62 with TIPS *vs*. 114 without TIPS). Patients in the no-TIPS group developed postoperative ACLF significantly more often than patients in the TIPS group (28 days: n = 33 [29%] *vs*. n = 6 [10%], *p* = 0.004; and 90 days: n = 36 [31%] *vs*. n = 9 [15%], *p* = 0.016) (Fig. S2). A competing risk analysis was not performed because of the small number of liver transplantations (only 1 and 2 events within 90 days and 12 months, respectively).

The distribution of ACLF grades and organ failures is presented in Table S4. There was a trend of higher ACLF grades in the no-TIPS group than in the TIPS group, although it was not statistically significant. Acute kidney injury was present in almost all patients. Precipitating events in the TIPS group were mostly associated with infections (83%). The no-TIPS group had a significantly higher proportion of unknown precipitating events compared with the TIPS group (60 *vs*. 0%, *p* = 0.014) (Table S4).

### Predictors of postoperative ACLF development

Cox regression analysis showed that in the no-TIPS group, the risk of 28- and 90-day ACLF development is increased more than 3-fold. Moreover, CLIF-C AD was an independent predictor of ACLF development 28 and 90 days after surgery (Table 2 and Table S5A). With MELD instead of CLIF-C AD included into the multivariate analysis, MELD and surgery without preoperative TIPS remained as predictors for 28- and 90-day ACLF development with hazard ratios (HRs) of 4.6 and 3.7, respectively (Table 3 and Table S5B). Univariate analysis of surgery with liver involvement and the extensiveness of surgery were not statistically significant. However, visceral surgery was significantly associated with ACLF development for both 28 and 90 days in the multivariate model (Tables S2A and S2B and Tables S5A and S5B). Stratified by the sub-cohorts of visceral and non-visceral surgery, multivariate analysis showed that surgery without preoperative TIPS is a predictor for 90-day ACLF development in patients undergoing visceral surgery but not non-visceral surgery (Tables S6A and S6B).

**Table 2:** **Univariate and multivariate Cox regression analysis for ACLF development within 90 days after surgery**.

	Univariate regression				Multivariate regression			
	*p* value	HR	95% CI		*p* value	HR	95% CI	
			Lower	Upper			Lower	Upper
Age	0.426	1.018	0.974	1.064	–	–	–	–
Aetiology	0.236	1.933	0.650	5.747	–	–	–	–
BMI	0.313	1.077	0.932	1.244	–	–	–	–
ASA score	0.755	0.881	0.397	1.956	–	–	–	–
***CLIF-C AD***	*0.020*	*1.077*	*1.012*	*1.146*	**0.016**	**1.085**	**1.015**	**1.159**
CRP	0.593	1.007	0.980	1.035	–	–	–	–
*Albumin*	*0.144*	*0.944*	*0.873*	*1.020*	–	–	–	–
***Visceral/non-visceral***	*0.124*	*2.038*	*0.822*	*5.051*	**0.034**	**2.763**	**1.083**	**7.051**
Liver involvement∗	0.28	1.62	0.67	3.92	–	–	–	–
Extensive/small†	0.14	0.49	0.19	1.25	–	–	–	–
Emergency/elective	0.46	1.51	0.51	4.49	–	–	–	–
Ascites	0.031	1.840	1.059	3.198	–	–	–	–
HE	0.730	0.049	‡	‡	–	–	–	–
MELD	0.027	1.190	1.020	1.388	–	–	–	–
Child-Pugh score	0.193	1.281	0.882	1.859	–	–	–	–
***Surgery without TIPS***	*0.029*	*2.869*	*1.113*	*7.398*	**0.016**	**3.256**	**1.248**	**8.499**

*Italic*—entered in multivariate regression model; **bold**—significant in multivariate regression analysis.

ACLF, acute-on-chronic liver failure; ASA, American Society of Anesthesiologists; BMI, body-mass index; CLIF-C-AD, Chronic Liver Failure Consortium–Acute decompensation; CRP, C-reactive protein; HE, hepatic encephalopathy; HR, hazard ratio; MELD, model for end-stage liver disease; TIPS, transjugular intrahepatic portosystemic shunt.

∗ All abdominal surgeries, where the liver was touched or mobilised by the operating surgeons or surgical instruments, were classified as visceral surgery with liver involvement.

† Surgeries with a duration over 90 min were defined as extensive.

‡ No clinical events.

**Table 3:** **Univariate and multivariate Cox regression analysis for ACLF development within 90 days after surgery with MELD instead of CLIF-C AD score**.

	Univariate regression				Multivariate regression			
	*p* value	HR	95% CI		*p* value	HR	95% CI	
			Lower	Upper			Lower	Upper
*Age*	0.426	1.018	0.974	1.064	–	–	–	–
*Aetiology*	0.236	1.933	0.650	5.747	–	–	–	–
BMI	0.313	1.077	0.932	1.244	–	–	–	–
ASA score	0.755	0.881	0.397	1.956	–	–	–	–
CLIF-C AD	0.020	1.077	1.012	1.146	–	–	–	–
CRP	0.593	1.007	0.980	1.035	–	–	–	–
***Albumin***	*0.144*	*0.944*	*0.873*	*1.020*	**0.050**	**0.915**	**0.837**	**1.000**
***Visceral/non-visceral***	*0.124*	*2.038*	*0.822*	*5.051*	**0.015**	**3.211**	**1.257**	**8.202**
Liver involvement∗	0.28	1.62	0.67	3.92	–	–	–	–
Extensive/small†	0.14	0.49	0.19	1.25	–	–	–	–
Emergency/elective	0.46	1.51	0.51	4.49	–	–	–	–
Ascites	0.031	1.840	1.059	3.198	–	–	–	–
HE	0.730	0.049	‡	‡	–	–	–	–
***MELD***	*0.027*	*1.190*	*1.020*	*1.388*	**0.003**	**1.353**	**1.107**	**1.652**
Child-Pugh score	0.193	1.281	0.882	1.859	–	–	–	–
***Surgery without TIPS***	*0.029*	*2.869*	*1.113*	*7.398*	**0.008**	**3.651**	**1.393**	**9.567**

*Italic*—entered in multivariate regression model; **bold**—significant in multivariate regression analysis.

ACLF, acute-on-chronic liver failure; ASA, American Society of Anesthesiologists; CLIF-C AD, Chronic Liver Failure Consortium–Acute Decompensation; CRP, C-reactive protein; HE, hepatic encephalopathy; HR, hazard ratio; MELD, model for end-stage liver disease; TIPS, transjugular intrahepatic portosystemic shunt.

∗ All abdominal surgeries, where liver was touched or mobilised by the operating surgeons or surgical instruments, were classified as visceral surgery with liver involvement.

† Surgeries with a duration over 90 min were defined as extensive.

‡ No clinical events.

Sensitivity analysis shows an E-value of 3.902, which indicates that the observed HR of 3.256 for ACLF development in the absence of TIPS at surgery could be explained away by an unmeasured confounder that is associated with both the presence of TIPS and ACLF development by a risk ratio of 3.9-fold each, above and beyond the measured confounders, but weaker confounding could not do so.

### Identification of high-risk patients

ROC analysis was performed for CLIF-C AD with 90-day ACLF development as the endpoint for the TIPS and no-TIPS groups. The results show a significant AUC (Harrell’s c 0.69; 95% CI 0.534–0.876) for the no-TIPS group (*p* = 0.041), whereas it does not for the TIPS group (Harrell’s c 0.537; 95% CI 0.256–0.817; *p* = 0.143). A CLIF-C AD cut-off of 45 in the no-TIPS group was chosen according to the Youden index. In patients with CLIF-C AD ≤45, no significant difference in the development rate of ACLF between the TIPS and no-TIPS groups could be detected (*p* = 0.610) (Fig. S3). However, in patients with CLIF-C AD >45, patients with TIPS showed a significantly lower rate of ACLF development after surgery (*p* = 0.015) (Fig. 2C). Adjusted for MELD, a MELD cut-off of ≥10 was identified by the Youden index for the identification of high-risk patients (Harrell’s c 0.660 for all patients; 95% CI 0.534–0.785; *p* = 0.027) (Fig. S4A and S4B).

### Postoperative outcome

The median duration of postoperative hospital stay was 11 days in both groups (11 [1–64] days in the TIPS *vs*. 11 [1–44] days in the no-TIPS group, *p* = 0.94). Patients in the no-TIPS group had significantly higher rates of unplanned readmissions to the intensive care unit owing to postoperative complications compared with the TIPS group (n = 21 [47%] *vs*. n = 11 [24%], *p* = 0.03). Within 90 days after surgery, patients in the TIPS group showed significantly lower rates of ascites development compared with those in the no-TIPS group (n = 15 [33%] *vs*. n = 25 [56%], *p* = 0.04). Postoperative blood transfusions were significantly more needed in patients of the no-TIPS group than in those of the TIPS group (n = 20 [44%] *vs.* n = 11 [24%], *p* = 0.05) (Table 4). Of note, there was no significant difference in the development of postoperative episodes of HE (TIPS: n = 5 [11%] *vs*. no-TIPS: n = 4 [9%], *p* = 0.73) and postoperative infections (TIPS: n = 13 [29%] *vs*. no-TIPS: n = 19 [42%], *p* = 0.19) between the groups (Table 4).

**Table 4:** **Postoperative complications within 90 days after surgery**.

	TIPS (n = 45)	No TIPS (n = 45)	*p* value
Duration of postoperative hospital stay (days)	11(1–64)	11 (1–44)	0.94
Postoperative stay at ICU	11 (24%)	21 (47%)	0.03
Postoperative ascites	15 (33%)	25 (56%)	0.04
Overt HE	5 (11%)	4 (9%)	0.73
Postoperative infection	13 (29%)	19 (42%)	0.19
Intraoperative and/or postoperative blood transfusion	11 (24%)	20 (44%)	0.05

Non-parametric testing was used to compare the groups, Mann-Whitney U test for comparison between continous variables and Chi-squared test for comparison between categorical variables. HE, hepatic encephalopathy; ICU, intensive care unit; TIPS, transjugular intrahepatic portosystemic shunt.

### Postoperative 1-year mortality

In total, 25 (28%) patients died within 1 year after surgery. Patients in the no-TIPS group had significantly higher 1-year mortality than those in the TIPS group (n = 17 [38%] *vs*. n = 8 [18%], *p* = 0.023) as shown in the survival curve (Fig. 2B). The most common cause of death was ACLF (n = 21), accounting for 84% of all deaths. The causes of death (ACLF *vs*. non-ACLF related) were not significantly different (Table S7).

Cox regression analysis revealed CLIF-C AD and surgery without preoperative TIPS as independent predictors of 1-year mortality. The type of surgery had no significant impact on the survival rate within 1 year (Table 5).

**Table 5:** **Univariate and multivariate Cox regression analysis for 1-year mortality after surgery**.

	Univariate regression				Multivariate regression			
	*p* value	HR	95% CI		*p* value	HR	95% CI	
			Lower	Upper			Lower	Upper
*Age*	0.146	1.032	0.989	1.078	–	–	–	–
*Aetiology*	0.212	1.868	0.701	4.981	–	–	–	–
BMI	0.710	0.973	0.844	1.123	–	–	–	–
ASA score	0.107	1.863	0.874	3.973	–	–	–	–
***CLIF-C AD***	*0.002*	*1.097*	*1.035*	*1.163*	**0.006**	**1.098**	**1.028**	**1.173**
*CRP*	*0.040*	*1.023*	*1.001*	*1.046*	–	–	–	–
*Albumin*	*0.006*	*0.897*	*0.830*	*0.970*	–	–	–	–
Visceral/non-visceral	0.38	1.43	0.64	3.18	–	–	–	–
Liver involvement∗	0.81	1.11	0.48	2.58	–	–	–	–
Extensive/small†	0.75	0.88	0.39	1.95	–	–	–	–
Emergency/elective	0.89	1.08	0.37	3.15	–	–	–	–
MELD	0.003	1.266	1.082	1.482	–	–	–	–
Child-Pugh score	0.046	1.428	1.007	2.024	–	–	–	–
***Surgery without TIPS***	*0.029*	*2.554*	*1.102*	*5.922*	**0.009**	**3.320**	**1.356**	**8.128**

*Italic*—entered in multivariate regression model; **bold**—significant in multivariate regression analysis.

ASA, American Society of Anesthesiologists; CLIF-C AD, Chronic Liver Failure Consortium–Acute Decompensation; CRP, C-reactive protein; HR, hazard ratio; MELD, model for end-stage liver disease; TIPS, transjugular intrahepatic portosystemic shunt.

∗ All abdominal surgeries, where the liver was touched or mobilised by the operating surgeons or surgical instruments, were classified as visceral surgery with liver involvement.

† Surgeries with a duration over 90 min were defined as extensive.

## Discussion

This study is the first to evaluate the postoperative development of ACLF between patients with cirrhosis with and without preoperative TIPS. It shows that the rate of ACLF development is significantly lower in patients with TIPS than in those without. This suggested effect seems to be more pronounced in high-risk patients with CLIF-C-AD scores above 45.

Surgical procedures in patients with cirrhosis are associated with a high complication rate despite advancements in surgical techniques and medical management and carry a mortality rate ranging from 10 to 57%.[18], [19], [20] Preoperative risk stratification in clinical practice is done according to traditional established scores such as Child-Pugh or MELD. Recently, the Veterans Outcomes and Costs Associated with Liver Disease (VOCAL)-Penn score was established with excellent prediction for postoperative mortality risk of different surgery types.^21^ Among the risk factors for all patients such as the ASA score, portal hypertension has been shown as 1 of the main predictors of fatal postoperative outcome.^22^ Owing to splanchnic vasodilation and portosystemic shunting, patients with portal hypertension have reduced hepatic blood flow, predisposing them to hypoperfusion of the liver during surgery, which can subsequently result in liver failure.^19^^,^^23^^,^^24^ Moreover, in a recent study, it was shown that HVPGs of >16 and ≥20 before surgery independently identify high-risk patients.^15^

The high perioperative risk in patients with cirrhosis and portal hypertension may preclude them from undergoing surgical procedures, which may be curative or improve quality of life. Thus, some smaller studies evaluated the feasibility of surgery in those patients, suggesting a benefit of decompression of portal pressure for postoperative complications associated with AD and outcome. Current data indicate that lowering of HVPG by TIPS before surgery may indeed increase the feasibility of planned surgeries in 52–85% of the patients.[25], [26], [27], [28] However, only 2 studies addressed postoperative outcome in patients with TIPS compared with those without.^26^^,^^29^ Unfortunately, the cohorts were matched neither for ASA score nor for liver function, resulting in significantly different Child-Pugh scores between the compared groups and not showing a significant difference in postoperative survival. Our study’s approach is unique in 2 ways: Firstly, it is the first to assess ACLF as the primary outcome. Secondly, the study results are based on a 1:1 matched cohort, which is controlled for all of the known confounders such as liver function (MELD and Child-Pugh), ASA score, type of surgery, age, sex, and aetiology of liver cirrhosis. Thus, our study is well controlled for confounders that could influence the results.

However, some unknown confounders may not be ruled out. Recently, TIPS has been shown to improve sarcopaenia and body composition,[30], [31], [32], [33] which is not only a risk factor for patients with liver cirrhosis in general but also for surgical procedures.[34], [35], [36] Even though our study did not specifically control for sarcopaenia, the BMI, as a surrogate of body composition, was not significantly different in our study. However, we acknowledge that BMI does not represent sarcopaenia or body composition in patients with ascites. Patients with TIPS showed less ascites than those without and thus may have better body composition and nutritional reserve and have lower rates of sarcopenia with similar BMI as compared with patients without TIPS. This would be in line with the known obesity paradox, which has been also noted in patients undergoing surgery.[37], [38], [39], [40], [41], [42] Thus, a potential influence on the observed difference in outcome cannot be ruled out. Although this should be further investigated, it is beyond the scope of this study. Moreover, the optimal rate of reduction of portosystemic pressure gradient should be explored in future prospective studies. Recent studies suggest beneficial effects of the use of smaller stent diameter.[43], [44], [45]

When looking at sub-cohorts, analyses reveal that surgery without preoperative TIPS is a predictor for ACLF development in visceral but not non-visceral surgeries. Many studies have shown that abdominal hepatic and non-hepatic surgeries are at an increased risk of poor outcome and that the type of surgery constitutes a major predictor in patients with cirrhosis.[46], [47], [48] Elevated HVPG levels before surgery seem to predict outcomes especially in patients undergoing extrahepatic abdominal or open chest surgery. Moreover, animal models showed elevated portal pressure after extrahepatic visceral surgery.^49^ These data suggest that increased postoperative mortality might be associated with aggravated portal hypertension in visceral surgery. Recent studies have been focussing on the heterogeneous postoperative risk in patients with cirrhosis and discuss an overprediction of mortality for certain subgroups. The recently established VOCAL-Penn score, differentiating between different types of abdominal, major orthopaedic, or open chest surgery may substantially improve postoperative mortality predictions in patients with cirrhosis.^21^^,^^47^ In this prediction model, major orthopaedic surgery and vascular surgery were not significantly associated with outcome. In our study, major orthopaedic and vascular surgeries represent the majority of surgeries categorised into non-visceral surgery. However, data on the influence of surgery types regarding ACLF as an endpoint are scarce. Moreover, the patient number in our study is small; thus, conclusions concerning the effect of preoperative TIPS on ACLF in different surgery categories should be considered with caution and be addressed in larger multicentric studies.

In our study, most of the deaths were related to ACLF. This is well in line with the high mortality rate of ACLF reported in the current literature.^7^^,^^9^ Moreover, TIPS was suggested to be beneficial to very sick patients with cirrhosis with acute variceal bleeding and presence of ACLF regarding mortality and rebleeding rates.^13^ Thus, our results, showing the highest effect of TIPS on ACLF development in patients with high CLIF-C-AD scores above 45, are further underlining the robustness of our data in the context of the current literature.

Despite the well-characterised cohort, our study has several limitations. This is a monocentric, retrospective study, which limits the generalisability of the results. Moreover, the study describes a relatively small cohort. Nevertheless, it represents the largest reported cohort of its kind in the current literature and is well controlled (by strict matching criteria) for known confounders of ACLF development and mortality. Moreover, our results are confirmed and strengthened by an internal validation cohort with more permissive 1:2 matching criteria, which allowed us to include almost all patients with preoperative TIPS. Therefore, the number of excluded patients of eligible study patients in the TIPS group was low (11 *vs*. 36%), which reduces the risk of selection bias. ACLF was diagnosed retrospectively; thus, punctual mis-grading of organ failure cannot be ruled out. However, information on the components of ACLF was retrieved from detailed digitalised patient data. Data of HVPG measurements before surgery were not routinely acquired but should be evaluated further in larger studies. However, surrogate parameters of portal hypertension such as varices status, platelet count, and spleen size were comparable. Another important factor for outcome is active alcohol misuse. Comprehensive anamnesis about ongoing alcohol misuse was not available in this retrospective dataset. However, we did not detect persistent harmful alcohol abuse at the time of surgery according to the National Institute on Alcohol Abuse and Alcoholism (NIAAA) criteria. Finally, the TIPS group did not specifically receive TIPS for the surgical procedure; thus, an allocation bias of the patients with TIPS cannot be ruled out. However, in a real-life clinical setting, many patients eligible for TIPS do not receive TIPS in the context of acute variceal bleeding, because of TIPS insertion not being possible in every hospital and patients missing the window of opportunity. This underlines that the data we are presenting are representative.^6^

In conclusion, this study suggests for the first time that patients with preoperative TIPS have lower rates of postoperative ACLF development compared with those without. The strongest effect is observed in patients undergoing visceral surgery and those with a CLIF-C AD score above 45.

## Financial support

JC is funded by grants from the Else-Kroener Fresenius Foundation (2014_Kolleg.05) and the BONFOR research program of the 10.13039/501100008131University of Bonn (grant ID 2019-2-08). JT is supported by grants from the 10.13039/501100001659Deutsche Forschungsgemeinschaft (SFB TRR57 to P18, CRC 1382 A09), 10.13039/100010661European Union’s Horizon 2020 Research and Innovation Programme (Galaxy, No. 668031; MICROB-PREDICT, No. 825694; and DECISION, No. 847949) and Societal Challenges–Health, Demographic Change and Wellbeing (No. 731875), and 10.13039/100008050Cellex Foundation (PREDICT). MP is funded by the Ernst-und-Berta Grimmke Foundation (No. 5/19) and the BONFOR research program of the 10.13039/501100008131University of Bonn (grant ID 2020-2A-07 and 2021-2A-07) and by the 10.13039/501100001659Deutsche Forschungsgemeinschaft (DFG, German Research Foundation) under Germany’s Excellence Strategy–EXC2151–390873048. The funders had no role in study design, data collection and analysis, decision to publish, or preparation of the manuscript.

## Authors’ contributions

Study concept and design: JT, MP. Study supervision: JT, MP. Acquisition of data: JC, PH, NB, PL. Analysis of data: JC, PH, NB, PL, JT, MP. Interpretation of data: JC, PH, NB, PL, JT, MP, SM, JD, DK, CJ, CM. Statistical analysis: JC, PH. Administrative, technical, and material support: JCK, CPS, JT, MP. Drafting of the manuscript: JC, PH, JT, MP. Critical revision of the manuscript regarding important intellectual content: NB, PL, SM, JD, DK, CJ, CM, JCK, CPS, JT, MP. Final approval of the version of the manuscript to be published: JT, MP

## Data availability statement

Source data are not openly available because of GDPR restrictions but can be requested via the corresponding author.

## Conflicts of interest

The authors have no conflicts of interest. Please refer to the accompanying ICMJE disclosure forms for further details.
