# Supplementary material for: Preoperative TIPS prevents the development of postoperative acute-on-chronic liver failure in patients with high CLIF-C AD score
Source: JHEP Rep. 2022 Jan 21;4(3):100442. doi: 10.1016/j.jhepr.2022.100442 (PMC8844300; doi:10.1016/j.jhepr.2022.100442)
Supplement: Multimedia component 1 [file mmc1.pdf]

Preoperative TIPS prevents the development of postoperative acute-on-chronic liver failure in patients with high CLIF-C AD score

Johannes Chang, Pauline Höfer, Nina Böhling, Philipp Lingohr, Steffen Manekeller, Jörg C. Kalff, Jonas Dohmen, Dominik J. Kaczmarek, Christian Jansen, Carsten Meyer, Christian P. Strassburg, Jonel Trebicka, Michael Praktiknjo

Table of contents

Fig. S1 2

Fig. S2 3

Fig. S3 4

Fig. S4 5

Table S1 7

Table S2 8

Table S3 10

Table S4 11

Table S5 12

Table S6 14

Table S7 15

Fig. S1. Kaplan-Meier plots showing probability of 28-day ACLF development for patients undergoing surgery in TIPS vs. no-TIPS group.

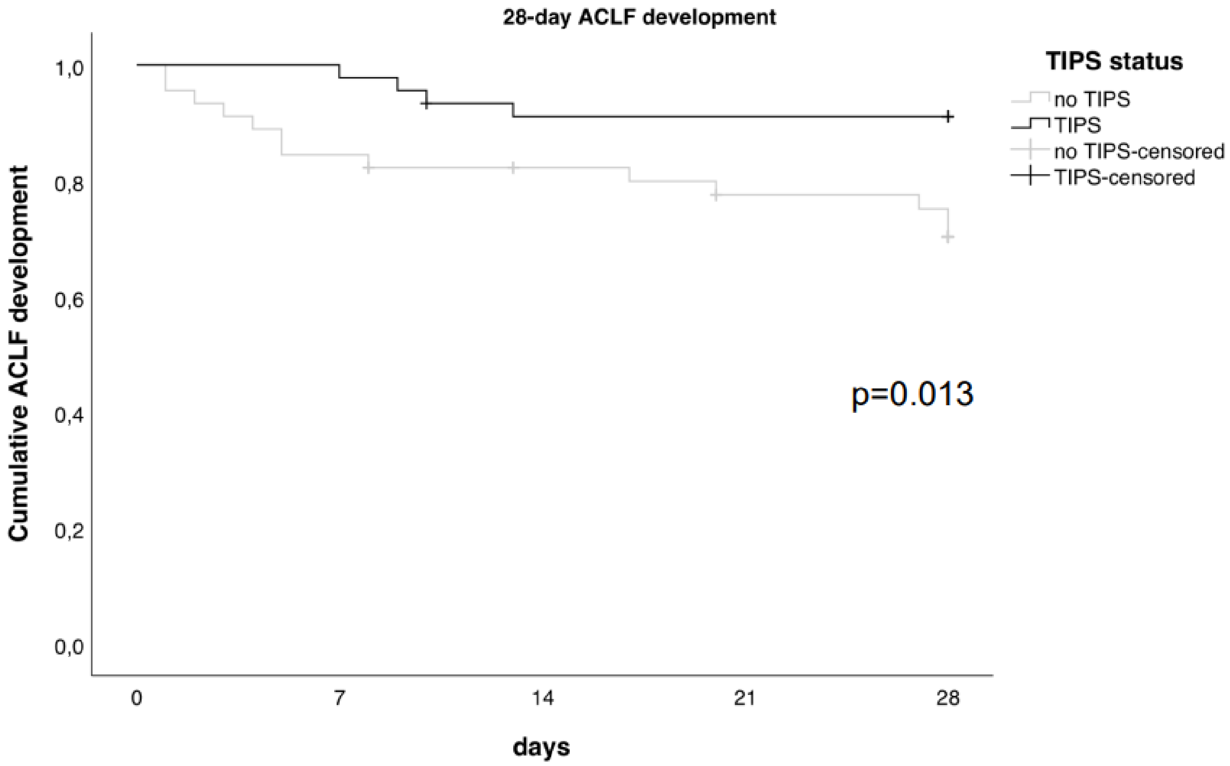

Patients at risk

No TIPS	45	38	35	32	31
TIPS	45	44	40	40	40

Probability of 28-day ACLF development calculated according to log-rank test for patients in the TIPS and no-TIPS group (n=90). Level of significance *p=0.013.

Fig. S2. Kaplan-Meier plots showing probability of 90-day ACLF development for patients undergoing surgery in TIPS vs. no-TIPS group of internal validation cohort (62 with and 114 without preoperative TIPS).

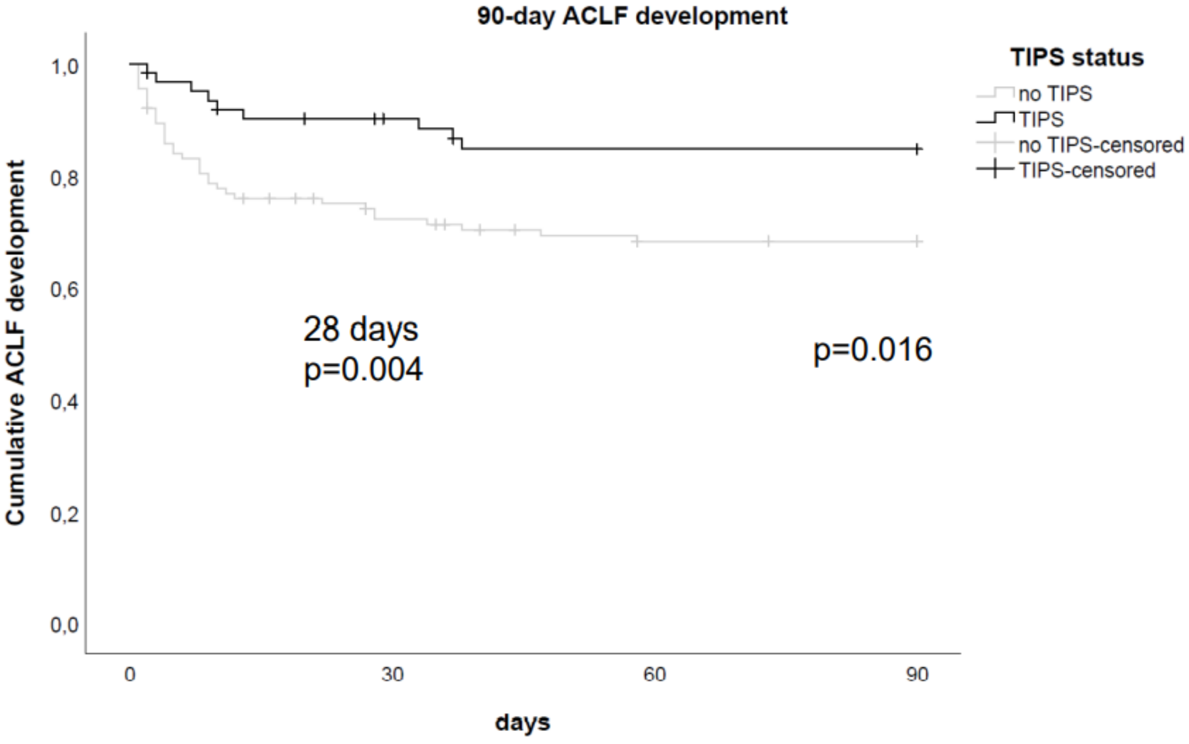

Patients at risk

No TIPS	114	77	68	67
TIPS	62	51	47	47

The initial cohorts were matched 1:2 with more permissive matching criteria, resulting in a validation cohort of 176 patients (62 with and 114 without preoperative TIPS). Probability of 90-day ACLF development calculated according to log-rank test for patients in the TIPS and no-TIPS group (n=176). Level of significance *p=0.016 and **p=0.004 for ACLF development within 28 days after surgery.

Fig. S3. Kaplan-Meier plots showing probability of 90-day ACLF development for patients undergoing surgery with a CLIF-C-AD ≤ 45 in TIPS vs. no-TIPS groups (n=39).

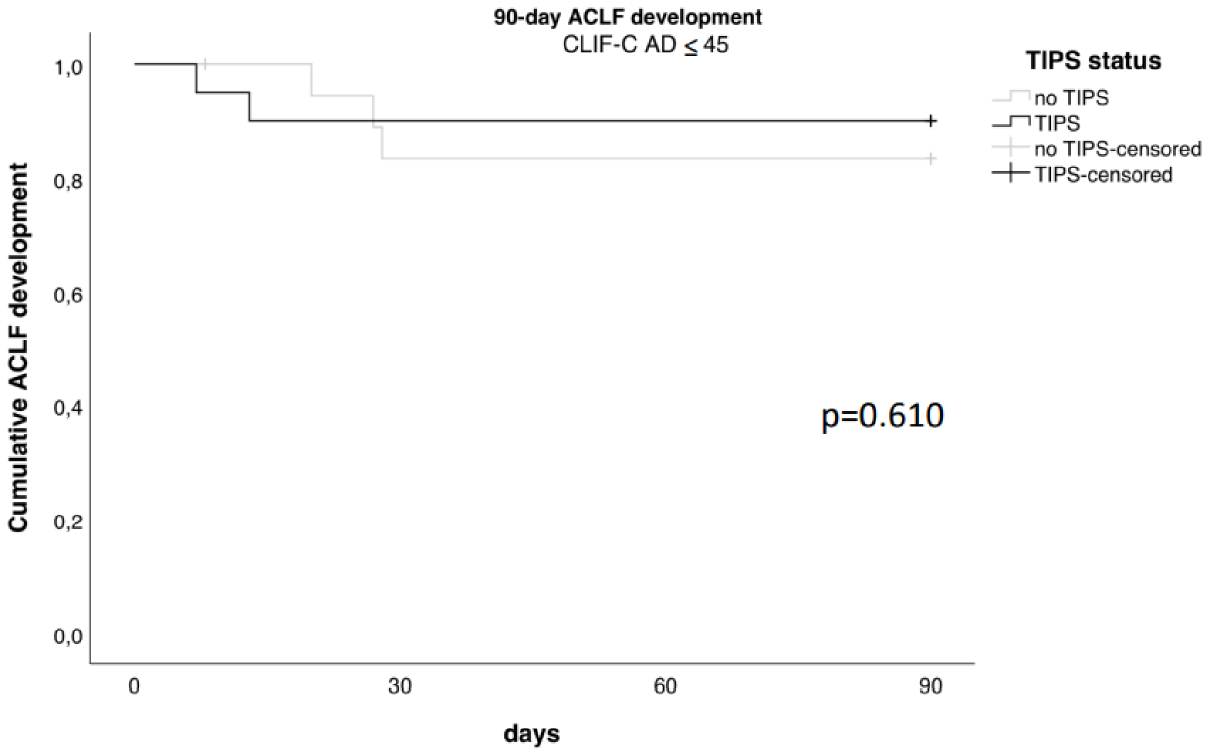

Patients at risk

No TIPS	19	15	15	15
TIPS	20	18	18	18

Probability of 90-day ACLF development calculated according to log-rank test for patients with a CLIF-C-AD ≤ 45 stratified to TIPS and no-TIPS groups (n=39). Level of significance p=0.610.

Fig. S4 Kaplan-Meier plots showing probability of 90-day ACLF development for patients undergoing surgery in TIPS and no-TIPS group.

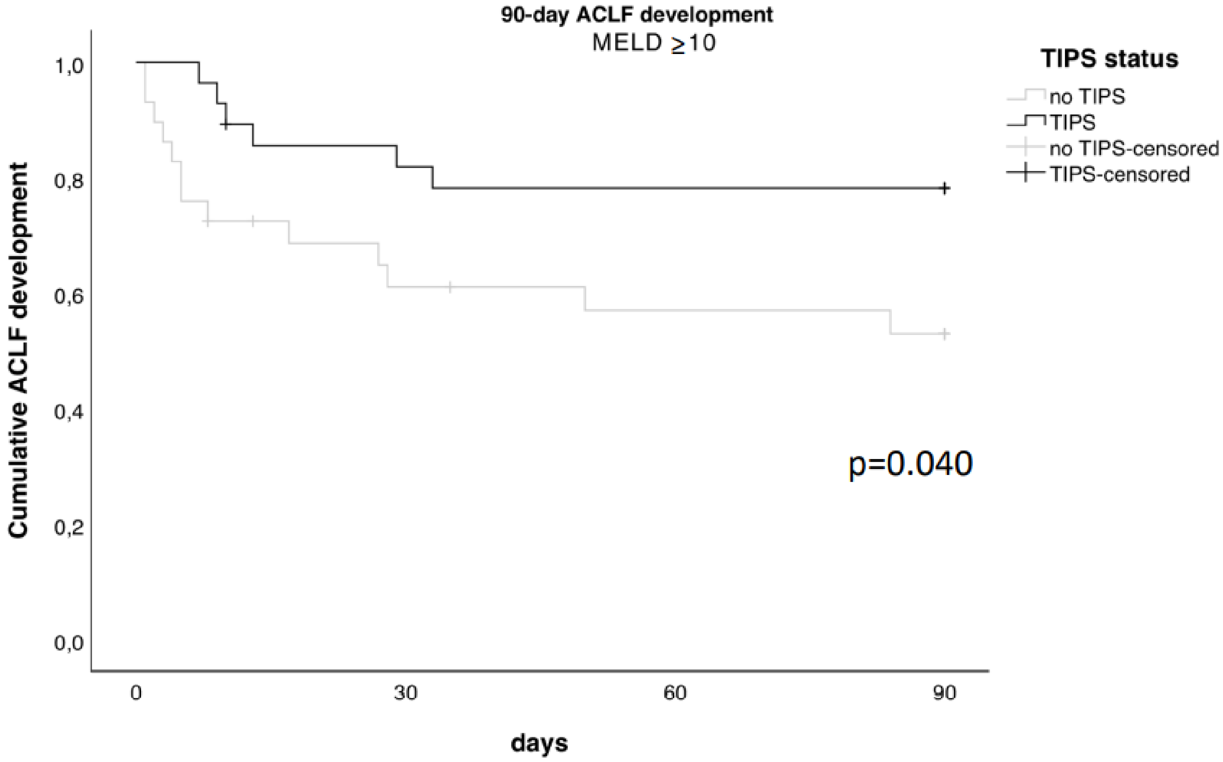

Patients at risk

No TIPS	29	16	14	13
TIPS	28	22	21	21

(A) Probability of 90-day ACLF development calculated according to log-rank test for patients with a MELD ≥ 10 stratified to TIPS and no-TIPS groups (n=33). Level of significance p=0.138.

Fig. S4B. Kaplan-Meier plots showing probability of 90-day ACLF development for patients undergoing surgery with a MELD < 10 in TIPS vs. no-TIPS groups (n=57).

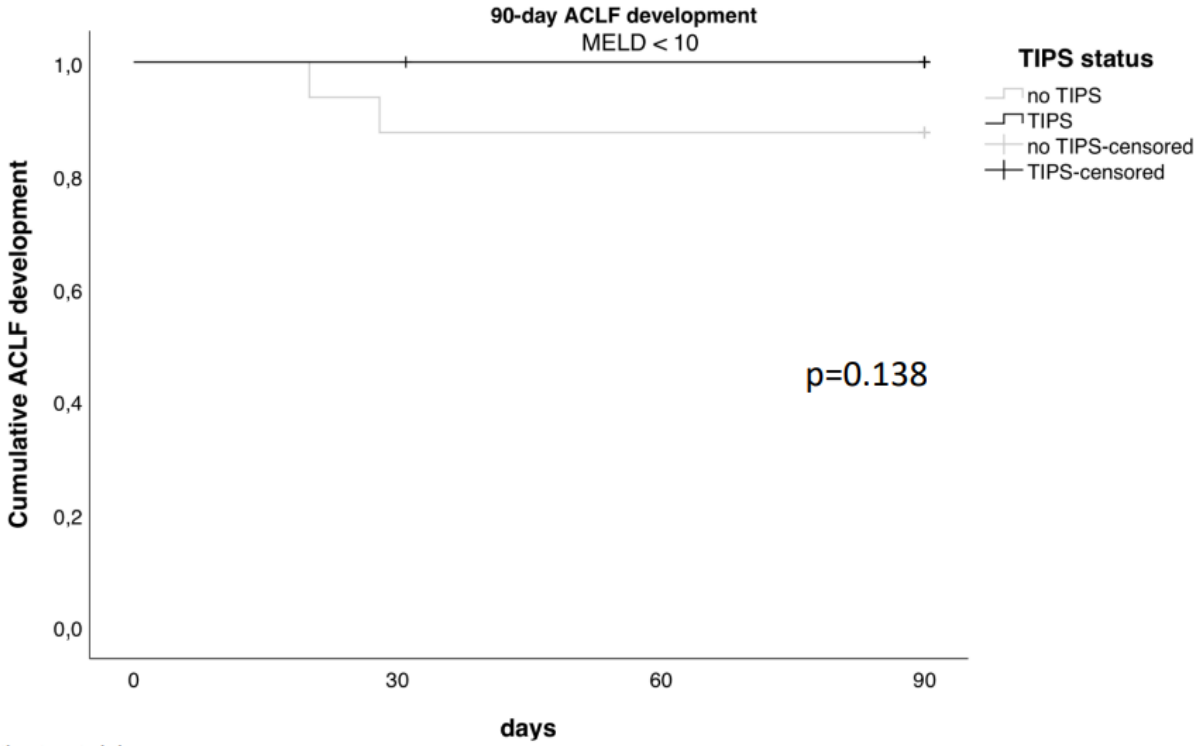

Patients at risk

No TIPS	16	14	14	14
TIPS	17	17	16	16

(B) Probability of 90-day ACLF development calculated according to log-rank test for patients with a MELD < 10 stratified to TIPS and no-TIPS groups (n=57). Level of significance *p=0.040.

Table S1. Cohort characteristics of unmatched cohort.

	Parameters at baseline	Unmatched patients (n=25)
General conditions	Age (years)	58 (30-74)
	Sex (male/female)	14/11 (56/44%)
	Etiology (alcohol/viral hepatitis/other)	15/5/5 (60/20/20%)
	BMI	25.7 (19-39)
Baseline scores	MELD score	14 (6-24)
	Child-Turcotte-Pugh class A/B/C	8/16/1 (32/64/4%)
	CLIF-C AD score	47 (39-59)
	Ascites	1 (4%)
Baseline laboratory	Sodium [mmol/l]	138 (127-144)
	Potassium [mmol/l]	4.09 (2.99-5.13)
	Creatinine [mg/dl]	0.89 (0.49-1.84)
	Bilirubin [mg/dl]	2.5 (0.40-4.90)
	ALT[U/l]	28 (9-215)
	AST [U/l]	43 (26-187)
	Albumin [g/dl]	33 (22.5-43)
	Gamma-GT [U/l]	167 (21-464)
	Alkaline phosphatase [U/l]	183 (77-279)
	INR	1.3 (1-1.9)
	CRP [mg/l]	23.4(1.5-48.3)
	Hb [g/dl]	10.6 (7.6-14.4)
	WBC [10*3/µl]	5.68 (2.74-12.5)
	Platelets [/µl]	99 (46-211)
Surgery	Non-visceral / visceral	17/8 (68/32%)
	Emergency / elective	3/22 (12/88%)
	ASA score (2/3/4)	2/20/3 (8/80/12%)
	Spleen diameter before TIPS [cm]	13.7 (8.8-23)
Medical history	History of ascites	17 (71%)
	History of GI-Bleeding	15 (63%)
	History of HE	8 (33%)
	Data are shown as median and ranges. Abbreviations: ALT, alanine transaminase; ASA, American Society of Anesthesiologists; AST, aspartate transaminase; BMI, body mass index; CLIF-C-AD, Chronic Liver Failure consortium acute decompensation score; CRP, c-reactive protein; gamma-GT, gamma glutamyl-transferase; GI, gastrointestinal; Hb, hemoglobin; HE, hepatic encephalopathy; INR, International normalized ratio; MELD, Model for End-stage Liver Disease; TIPS, transjugular intrahepatic portosystemic shunt; WBC, white blood cells	

Table S2A. Detailed list of surgeries.

Visceral (n=48)	n	Non-Visceral (n=42)	n
Hernia repair	17		
Cholecystectomy	11	Orthopedic surgery#	25
Liver resection	11	Vascular surgery§	6
Bowel resection*	4	Other$	11
Other+	5		

* jejunum resection; colon resection; sigma resection; rectum resection

+gynecological surgery: salpingo-ovariectomy, 3 x hysterectomy and/or salpingectomy; pancreaticojejunostomy

# wound debridement; kyphoplasty; spondylodesis; implantation hip prothesis; clavicular fracture; humerus fracture; adial fracture; partial pelvis resection; implantation external knee fixator; explantation knee prothesis; arthrodesis ankle joint; osteosynthesis acromioclavicular joint; decompression of the vertebral canal

§thrombendarteriectomy; aortic prothesis

$oral and maxillofacial surgery: mandibular fracture operation, jaw reconstruction; cardiothoracic surgery: pleurodesis, partial lung resection, aortic valve replacement; haematoma removal; lymph node excision; exstirpation of parathyroid gland

Table S2B. Surgery distribution between TIPS and No-TIPS group.

Type of surgery	TIPS (n=45)	No TIPS (n=45)	p
**Visceral**			
Hernia repair	11 (24%)	6 (13%)	0.181
Cholecystectomy	5 (11%)	6 (13%)	0.749
Liver resection	5 (11%)	6 (13%)	0.749
Bowel resection	2 (4%)	2 (4%)	1.000
Other (visceral)	1 (2%)	4 (9%)	0.170
**Non-Visceral**			
Orthopedic surgery	15 (33%)	10 (22%)	0.242
Vascular surgery	2 (4%)	4 (9%)	0.401
Other (non-visceral)	4 (9%)	7 (16%)	0.337

Table S3. General characteristics of validation cohort with more permissive matching criteria.

	Parameters at baseline	TIPS n=62	No TIPS n=114	p
General conditions	Age (years)	61.74 (± 9.06)	61.22 (± 11.37)	0.755
	Sex (male/female)	42/20 (68/32%)	84/30 (74/26%)	0.404
	Etiology (alcohol/viral hepatitis/other)	42/7/13 (68/11/21%)	64/8/42 (56/7/37%)	0.082
	BMI	26.3 (17.4-39)	26.3 (18-48)	0.767
Baseline scores	MELD score	12 (6-20)	10 (6-24)	0.201
	Child-Turcotte-Pugh A/B&C	16/46 (26/74%)	36/78 (32/68%)	0.424
	CLIF-C AD score	47 (29-64)	49 (29-72)	0.130
Baseline laboratory	Sodium [mmol/l]	139 (127-145)	138 (122-145)	0.244
	Creatinine [mg/dl]	1.00 (0.49-1.84)	1.00 (0.55-1.92)	0.947
	Bilirubin [mg/dl]	1.43 (0.32-4.85)	1.08 (0.29-4.69)	0.023
	ALT[U/l]	25 (9-333)	27 (6-152)	0.305
	AST [U/l]	41 (18-283)	38 (13-174)	0.489
	Albumin [g/dl]	31.9 (20.8-46.2)	31.7(17.9-43.4)	0.686
	INR	1.2 (0.9-1.9)	1.1 (0.9-2.2)	0.105
	CRP [mg/l]	9.2 (0.48-90.4)	11.9 (0.6-280.0)	0.160
	Hb [g/dl]	10.55 (7.60-16.00)	11.4 (5.8-17.0)	0.003
	WBC [10*3/µl]	5.3 (2.25-12.5)	6.64 (1.23-14.66)	0.127
	Platelets [/µl]	144 (25-336)	144 (23-388)	0.171
Surgery	Non-visceral / visceral	29/33 (47/53%)	48/66 (42/58%)	0.551
	Emergency / elective	10/52 (16/84%)	26/48 (23/77%)	0.294
	ASA score (2/3/4)	14/41/7 (23/66/11%)	34/67/13 (30/59/11%)	0.569
	Data are shown as median and ranges. Abbreviations: ALT, alanine transaminase; ASA, American Society of Anesthesiologists; AST, aspartate transaminase; BMI, body mass index; CLIF-C-AD, Chronic Liver Failure consortium acute decompensation score; CRP, c-reactive protein; gamma-GT, gamma glutamyl-transferase; GI, gastrointestinal; Hb, hemoglobin; INR, International normalized ratio; MELD, Model for End-stage Liver Disease; TIPS, transjugular intrahepatic portosystemic shunt; WBC, white blood cells			

Table S4. Grades, type of organ failures and precipitating events of postoperative ACLF within 90 days after surgery.

	TIPS (n=6)	No TIPS (n=15)	p
**ACLF Grade**			
Any grade	6 (13%)[$]	15 (33%)[$]	0.020
I	4 (67%)	6 (40%)	0.281
II	0 (0%)	6 (40%)	0.074
III	2 (33%)	3 (20%)	0.527
**Precipitating event**			
Infection	5 (83%)	6 (40%)	0.080
GI-Bleeding	1 (17%)	0 (0%)	0.114
Unkown	0 (0%)	9 (60%)	0.014
- Unknown with HRS	0 (0%)	1 (11%)[#]	
- Unknown, with HE	0 (0%)	4 (44%)[#]	
**Type of organ failure**			
Kidney	6 (100%)	13 (87%)	0.359
Lung	2 (33%)	3 (20%)	0.527
Brain	0 (0%)	3 (20%)	0.248
Circulation	2 (33%)	5 (33%)	1.000
Hemostasis	0 (0%)	4 (27%)	0.170
Liver	1 (17%)	3 (20%)	0.812

[$] percentage are in relation to the whole cohort (n=45 respectively)

[#] percentages are in relation to all unknowns (n=9)

ACLF, acute-on-chronic liver failure; GI, gastrointestinal; HE, hepatic encelopathy; HRS, hepatorenal syndrome; TIPS, transjugular intrahepatic portosystemic shunt

Table S5A. Univariate and multivariate Cox regression analysis for ACLF development within 28 days after surgery.

univariate regression					multivariate regression			
			95% CI				95% CI	
	p	HR	lower	upper	p	HR	lower	upper
Age	0.453	1.019	0.970	1.070				
Etiology	0.238	2.118	0.609	7.374				
ASA Score	0.414	0.706	0.306	1.628				
CLIF-C-AD	*0.047*	*1.072*	*1.001*	*1.148*	**0.035**	**1.083**	**1.006**	**1.165**
CRP	0.718	1.006	0.975	1.037				
Albumin	*0.381*	*0.963*	*0.885*	*1.048*				
Visceral/non-visceral	*0.037*	*3.295*	*1.074*	*10.112*	**0.013**	**4.258**	**1.352**	**13.412**
Liver involvement+	0.22	1.83	0.70	4.80				
Extensive/small$	0.09	0.38	0.12	1.16				
Emergency/elective	0.23	2.00	0.65	6.13				
Ascites	0.014	2.106	1.164	3.810				
HE	0.760	0.049	#	#				
MELD	0.092	1.157	0.977	1.370				
CTP score	0.486	1.154	0.771	1.729				
Surgery without TIPS	*0.023*	*3.653*	*1.191*	*11.208*	**0.015**	**4.071**	**1.318**	**12.577**

ACLF, acute-on-chronic liver failure; ASA, American Society of Anesthesiologists; BMI, body-mass index; CTP-Score, Child-Turcotte-Pugh-Score; CI, confidence interval; CLIF-C-AD, Chronic Liver Failure consortium acute decompensation score; CRP, c-reactive protein; HE, hepatic encephalopathy; HR, hazard ratio; MELD, Model for End-stage Liver Disease; TIPS, transjugular intrahepatic portosystemic shunt; *italic* – entered in multivariate regression model, **bold** – significant in multivariate regression analysis, # no clinical events

+all abdominal surgeries, where the liver was touched or mobilized by the operating surgeons or surgical instruments were classified as visceral surgery with liver involvement

$surgeries with a duration over 90 minutes were defined as extensive

Table S5B. Univariate and multivariate Cox regression analysis for ACLF development within 28 days after surgery with MELD instead of CLIF-C AD.

	**univariate regression**				**multivariate regression**			
			95% CI				95% CI	
	p	HR	lower	upper	p	HR	lower	upper
Age	0.453	1.019	0.970	1.070				
Etiology	0.238	2.118	0.609	7.374				
ASA Score	0.414	0.706	0.306	1.628				
CLIF-C-AD	0.047	1.072	1.001	1.148				
CRP	0.718	1.006	0.975	1.037				
Albumin	*0.381*	*0.963*	*0.885*	*1.048*				
Visceral/non-visceral	*0.037*	*3.295*	*1.074*	*10.112*	**0.006**	**4.998**	**1.572**	**15.892**
Ascites	0.014	2.106	1.164	3.810				
HE	0.760	0.049	#	#				
MELD	*0.092*	*1.157*	*0.977*	*1.370*	**0.013**	**1.310**	**1.059**	**1.620**
CTP score	0.486	1.154	0.771	1.729				
Surgery without TIPS	*0.023*	*3.653*	*1.191*	*11.208*	**0.009**	**4.573**	**1.465**	**14.268**

ACLF, acute-on-chronic liver failure; ASA, American Society of Anesthesiologists; BMI, body-mass index; CTP-Score, Child-Turcotte-Pugh-Score; CI, confidence interval; CLIF-C-AD, Chronic Liver Failure consortium acute decompensation score; CRP, c-reactive protein; HE, hepatic encephalopathy; HR, hazard ratio; MELD, Model for End-stage Liver Disease; TIPS, transjugular intrahepatic portosystemic shunt; *italic* – entered in multivariate regression model, **bold** – significant in multivariate regression analysis, # no clinical events

Table S6A. Univariate and multivariate Cox regression analysis for ACLF development within 90 days, only patients undergoing visceral surgery (n=48)

univariate regression					multivariate regression			
			95% CI				95% CI	
	p	HR	lower	upper	p	HR	lower	upper
Age	0.147	1.045	0.985	1.108				
Etiology	0.203	2.643	0.591	11.818				
CLIF-C-AD	0.057	1.087	0.998	1.185				
Albumin	0.027	0.891	0.804	0.987				
Surgery without TIPS	0.007	7.803	1.743	34.939	**0.007**	**7.803**	**1.743**	**34.939**

ACLF, acute-on-chronic liver failure; CI, confidence interval; CLIF-C-AD, Chronic Liver Failure consortium acute decompensation score; HR, hazard ratio; TIPS, transjugular intrahepatic portosystemic shunt; *italic* – entered in multivariate regression model, **bold** – significant in multivariate regression analysis

Table S6B. Univariate and multivariate Cox regression analysis for ACLF development within 90 days, only patients undergoing non-visceral surgery (n=42)

univariate regression					multivariate regression			
			95% CI				95% CI	
	p	HR	lower	upper	p	HR	lower	upper
Age	0.676	0.985	0.920	1.055				
Etiology	0.700	0.986	0.919	1.058				
CLIF-C-AD	0.144	1.076	0.975	1.188				
Albumin	0.932	0.994	0.874	1.131				
Surgery without TIPS	0.650	0.707	0.158	3.159				

ACLF, acute-on-chronic liver failure; CI, confidence interval; CLIF-C-AD, Chronic Liver Failure consortium acute decompensation score; HR, hazard ratio; TIPS, transjugular intrahepatic portosystemic shunt; *italic* – entered in multivariate regression model, **bold** – significant in multivariate regression analysis

Table S7. Causes of death

Cause of death<br>1-Year	Total<br>(N=25)	No TIPS<br>(N=17)	TIPS<br>(N=8)	p
ACLF related death	21 (84%)	15 (88%)	6 (75%)	0.409
Non-ACLF related death<br>- HCC	4 (16%)	2 (12%)<br>1 (6%)	2 (25%)<br>1 (13%)	0.140

ACLF, acute-on-chronic liver failure; HCC, hepatocellular carcinoma; TIPS, transjugular intrahepatic portosystemic shunt
